# Structural abnormalities detected by knee magnetic resonance imaging are common in middle-aged subjects with and without risk factors for osteoarthritis

**DOI:** 10.1080/17453674.2018.1495164

**Published:** 2018-07-17

**Authors:** Jaanika Kumm, Aleksandra Turkiewicz, Fan Zhang, Martin Englund

**Affiliations:** 1 Department of Radiology, University of Tartu, Tartu, Estonia;; 2 Lund University, Faculty of Medicine, Department of Clinical Sciences Lund, Orthopaedics, Clinical Epidemiology Unit, Lund, Sweden;; 3 Clinical Epidemiology Research and Training Unit, Boston University School of Medicine, Boston, MA, USA

## Abstract

Background and purpose — Few data are available regarding structural changes present in knees without radiographically evident osteoarthritis (OA). We evaluated the prevalence of findings suggestive of knee OA by magnetic resonance imaging (MRI) in middle-aged subjects without radiographic OA with or without OA risk factors.

Patients and methods — 340 subjects from the Osteoarthritis Initiative, aged 45–55 years (51% women) with Kellgren–Lawrence grade 0 in both knees, who had 3T knee MR images were eligible. 294 subjects had risk factors and 46 were without risk factors. MR images were assessed using the MOAKS scoring system.

Results — At least 1 MR-detected feature was found in 96% (283/294) of subjects with risk factors and in 87% (40/46) of those without. Cartilage damage (82%), bone marrow lesions (60%), osteophytes (45%), meniscal body extrusion (32%), and synovitis–effusion (29%) were the most common findings in subjects with risk factors, while cartilage damage (67%), osteophytes (46%), meniscal body extrusion (37%), and bone marrow lesions (35%) were most common in subjects without. The prevalence of any abnormality was higher in subjects with OA risk factors than in subjects without (prevalence ratio adjusted for age and sex 1.3 [95% CI 1.1–1.6]), so was prevalence of subchondral cysts and bone marrow lesions. MR-detected structural changes were more frequent in patellofemoral joints.

Interpretation — Our findings highlight the great challenge in distinguishing pathological features of early knee OA from what could be considered part of “normal ageing.” Bone marrow lesions were more frequently found in subjects with multiple OA risk factors.

Recently, a high prevalence of magnetic resonance imaging (MRI) detected osteophytes, cartilage damage, and other OA-related pathological features have been identified in the tibiofemoral joint of knees without any evidence of OA on conventional radiographs (Hayes et al. 2005, Englund et al. 2008, Davies-Tuck et al. 2009, Javaid et al. 2010, Roemer et al. 2011, Guermazi et al. 2012, Hayashi et al. 2014, Sharma et al. 2014). This new knowledge has highlighted the difficulty in distinguishing true pathological features of early OA from those potentially related to what could be considered “normal ageing” of the joint.

There is an inevitable risk of interpreting incidental findings on MRI as related to early OA. It is therefore critical to gain further insights into the presence of anatomical alterations that could be considered suggestive of early OA in persons without radiographic evidence of knee OA to improve the understanding about the pre-radiographic stage of the disease.

Thus, our aim was to determine the prevalence of a broad spectrum of knee joint structural features on MRI in Osteoarthritis Initiative (OAI) participants (aged 45–55 years) who had radiographically normal knees (Kellgren and Lawrence grade 0 in both knees). Importantly, we stratified the study cohort into those having common risk factors for knee OA vs. those without risk factors. We report the structural changes in both the tibiofemoral and the patellofemoral joint and provide estimates of the prevalence of tibiofemoral and patellofemoral OA based on an MRI definition (Hunter et al. 2011a).

## Patients and methods

### Study sample

Subjects were sampled from the Osteoarthritis Initiative (OAI) database. Details for this multicenter longitudinal observational study are available for public access at http://www.oai.ucsf.edu/. Our criteria for sampling were: (i) age 45–55 years; (ii) no evidence of radiographic tibiofemoral OA of either knee at baseline based on the central readings (weight-bearing fixed-flexion posterior-anterior knee radiographs, Kellgren and Lawrence grade =0); and (iii) available knee MRIs at baseline (and at 24, 48, and 72 months’ follow-up) (the follow-up images were not used for the current study). All subjects that fulfilled the above criteria were selected for the study. The subjects were drawn from the OAI incidence, progression, and “non-exposed” reference cohort. The subjects in the incidence and progression cohort had risk factors for OA like obesity, previous knee trauma and/or surgery, family history of total knee joint replacement, presence of Heberden’s nodes and repetitive knee bending but were given Kellgren–Lawrence grade 0 at baseline in both knees when assessed by the central reading facility in Boston. The reference cohort had no such risk factors, and no pain, aching, or stiffness in either knee in the past year before the baseline examination.

340 subjects met the above criteria. Among them were 294 subjects with OA risk factors (278 and 16 subjects from the incidence and progression cohort, respectively) and 46 individuals without OA risk factors (the reference cohort).

### Knee joint radiography protocol

The standing fixed-flexion knee radiography protocol was used to assess OA in the tibiofemoral joints. All participants had bilateral standing knee films obtained in posterior-anterior projection with knees flexed to 20–30 degrees and feet internally rotated 10 degrees. The degree of knee flexion and foot rotation was fixed using a plexiglass positioning frame (SynaFlexer, Synarc, Inc, San Francisco, CA, USA).

### MRI protocol and assessment of knee joint structural abnormalities

The MR images of subjects’ knees were obtained at the 4 clinical study centers using Siemens Trio 3.0-Tesla MRI scanners and quadrature transmit–receive extremity radio-frequency coils as previously detailed (Kumm et al. 2016).

One radiologist (JK), who was blinded to subject characteristics, interpreted the images for osteophytes, cartilage damage, BMLs including cysts, meniscal tears, synovitis–effusion, Hoffa synovitis, ligament abnormalities, and popliteal cysts and scored these features semi-quantitatively according to the Magnetic Resonance Imaging Osteoarthritis Knee Score (MOAKS) (Hunter et al. 2011b). In MOAKS, the knee joint is divided into 14 articular sub-regions for scoring articular cartilage, into 15 sub-regions for scoring bone marrow lesions (BMLs) and into 12 regions for scoring osteophytes. In the present study, cartilage damage, and bone marrow lesions were scored in 14 sub-regions, excluding the tibial subspinous subregion. Also, for the purpose of this study, we only report findings from right knees.

For further analysis, we also divided knee joints into three different articular compartments—patellofemoral, medial, and lateral tibiofemoral.

Osteophytes were scored using a 0–3 scale (grade 0 = none; grade 1 = small; 2 = medium; 3 = large). Osteophytes were considered as present when MOAKS grade ≥1. Cartilage damage was graded for the size of cartilage loss as a percentage of surface area as related to the size of subregion surface; and for the percentage of loss in the subregion that is full-thickness loss using 0–3 scales. For the size of cartilage loss, the following grading system was used: 0 = none; 1 = < 10%; 2 = 10–75%; 3 = > 75% of sub-region of cartilage surface area; and for the percentage of full-thickness cartilage loss: 0 = none; 1 = < 10%; 2 = 10–75%; and 3 = > 75% of subregion of cartilage surface area. Cartilage damage graded as grade ≥1.0 was considered as having cartilage damage. Bone marrow lesions were scored as hyperintense signal within the trabecular bone on T2-weighted fat-suppressed images using a 0–3 scale: 0 = none; 1 = < 33%; 2 = 33–66%; and >66% of subregional volume. Subchondral cysts were assessed as a percentage of bone marrow lesion that is cyst using a 0–3 scale: 0 = none; 1 = < 33%; 2 = 33–66%; and 3 = > 66% of the lesion. We considered bone marrow lesions and cysts present if graded ≥1. Meniscal damage (meniscal tears and/or destruction) was assessed in the anterior horn, body, and posterior horn of the menisci. Meniscal tears were considered present if intra-meniscal increased signal communicated with the superior, inferior, or free edge of the meniscal surface on at least 2 consecutive images (De Smet et al. 2006). Another observer (FZ, orthopedic surgeon) measured meniscal body extrusion to the closest 0.1 mm using mid-coronal images and Sante DICOM Editor (64-bit) software (http://www.santesoft.com/win/sante-dicom-editor/sante-dicom-editor.html) (Zhang et al. 2016). Intra-observer reliability for meniscal measurements ranged from 0.6 to 1. We defined meniscal extrusion as extrusion = > 3mm. Synovitis-effusion was graded using a 0–3 scale: grade 0 = none; grade 1 = small; grade 2 = medium; grade 3 = large. Synovitis-effusion was present if graded ≥1. Hoffa synovitis and popliteal cysts were scored as absent/present.

30 randomly selected subjects’ knees were reassessed. Intra-observer reliability measured as kappa coefficients was as follows: 0.4 (CI 0.1–0.8) for the detection of osteophytes, 0.7 (CI 0.4–1.0) for synovitis–effusion, 0.9 (CI 0.8–1.0) for meniscal damage, and 0.8 (CI 0.5–1.0) for popliteal cysts. All knees included in the reliability assessment were graded consistently in both readings for the presence of cartilage damage and bone marrow lesions (kappa =1.0).

### Statistics

We calculated the prevalence of MRI features in right knees. As the individuals with risk factors were oversampled in the OAI cohort, to avoid bias we present separate estimates for those with and without risk factors. The results are presented as percentage with 95% confidence intervals (CI) calculated according to the Agresti–Coull method (Agresti and Coull 1998). We also determined the prevalence of MRI-defined knee OA based on the method described by Hunter et al. (2011a). To estimate the prevalence ratio among persons with and without OA risk factors we used a Poisson regression model with robust standard errors and general estimating equations to account for correlation between different features within the same knee. The model was adjusted for age and sex. Additionally, we used a Poisson regression model with robust standard errors to estimate adjusted (for age and sex) prevalence ratio of the specific features.

### Ethics, funding, and conflicts of interest

The OAI has been approved by the institutional review boards for the University of California, San Francisco and the four OAI clinical centers (University of Pittsburgh; Ohio State University; University of Maryland, Baltimore; Memorial Hospital of Rhode Island). All subjects have given informed consent to participate in the study.

JK reports a grant from the Osteoarthritis Research Society International (young investigator travel award). ME reports grants from the Swedish Research Council, Kock Foundations, the Swedish Rheumatology Association, Österlund Foundation, the Faculty of Medicine, Lund University, Sweden, and Governmental Funding of Clinical Research within the National Health Service (ALF). The funders had no role in study design, data collection and analysis, decision to publish, or preparation of the manuscript. The authors declare no conflicts of interest.

## Results

The mean (SD) age of study subjects, n = 340 (173; 51% women), was 50 years (3.0) (range 45–55) years, and the mean (SD) BMI was 27 (4.4) (18–32) (Table 1).

**Table 1.** Characteristics of the study participants. Values are frequency and (percentage) unless otherwise stated

	Subjects with OA risk factors (n = 294)	Reference cohort (n = 46)
Age, mean (SD)	50 (2.9)	50 (3.3)
Women	146 (50)	27 (59)
BMI ^a^:		
< 25	104 (35)	26 (57)
25–29	114 (39)	20 (44)
≥ 30	70 (24)	0 (0)
Family history of joint replacement**^b^**	53 (18)	0 (0)
Heberden’s nodes, either hand**^b^**	55 (19)	2 (4)
Engage in at least 1 knee bending activity**^b^**	203 (69)	19 (41)
Knee symptoms (pain, aching, or stiffness), either knee**^b^**	273 (93)	0 (0)

**^a^**Data on BMI (body mass index) is missing for 6 individuals.

**^b^** As assessed in eligibility interviews at baseline.

### Prevalence in subjects with risk factors for knee OA

283 (96%) subjects with knee OA risk factors but without radiographic knee OA had at least 1 abnormality present (Table 2). The most common findings were cartilage damage (82%, CI 77–86), bone marrow lesions (60%, CI 54–65), osteophytes (45%, CI 39–50), Hoffa synovitis (44%, CI 39–50), and subchondral cysts (41%, CI 35–46). MRI features like meniscal extrusions (23%, CI 19–28), synovitis–effusion (29%, CI 24–35), popliteal cysts (28%, CI 23–34), and meniscal damage (19%, CI 15–24) were also quite frequently encountered in these individuals with radiographically normal knees. The prevalence of ligamentous lesions was low (1%, CI 0–4).

**Table 2.** Prevalence of MRI structural abnormalities in the right knees of the study subjects with knee OA risk factors. Values are frequency and (percentage)

MRI feature	Overall (n = 294)	Women (n = 146)	Men (n = 148)	BMI ^a^		
				< 25 (n = 104)	25–29 (n = 114)	≥ 30 (n = 70)
Cartilage damage	240 (82)	125 (86)	115 (78)	80 (77)	94 (82)	61 (87)
Tibiofemoral	189 (64)	90 (62)	99 (67)	65 (63)	70 (61)	49 (70)
Tibiofemoral medial	120 (41)	55 (38)	65 (44)	48 (46)	46 (40)	23 (33)
Tibiofemoral lateral	127 (43)	60 (41)	67 (45)	43 (41)	45 (39)	34 (49)
Patellofemoral	199 (68)	109 (75)	90 (61)	64 (62)	80 (70)	52 (74)
Osteophytes	131 (45)	56 (38)	75 (51)	35 (34)	58 (51)	36 (51)
Tibiofemoral	56 (19)	28 (19)	28 (19)	21 (20)	20 (18)	14 (20)
Tibiofemoral medial	43 (15)	21 (14)	22 (15)	17 (16)	13 (11)	12 (17)
Tibiofemoral lateral	30 (10)	15 (10)	15 (10)	9 (9)	11 (10)	9 (13)
Patellofemoral	124 (42)	52 (36)	72 (49)	33 (32)	54 (47)	35 (50)
Bone marrow lesions	176 (60)	91 (62)	85 (57)	59 (57)	64 (56)	49 (70)
Tibiofemoral	91 (31)	40 (27)	51 (34)	34 (33)	32 (28)	22 (31)
Tibiofemoral medial	54 (18)	23 (16)	31 (21)	24 (23)	17 (15)	13 (19)
Tibiofemoral lateral	43 (15)	18 (12)	25 (17)	11 (11)	18 (16)	11 (16)
Patellofemoral	138 (47)	75 (51)	63 (43)	43 (41)	56 (49)	38 (54)
Subchondral cysts	119 (40)	67 (46)	52 (35)	38 (37)	46 (40)	32 (46)
Tibiofemoral	51 (17)	24 (16)	27 (18)	17 (16)	19 (17)	13 (19)
Tibiofemoral medial	31 (11)	14 (10)	17 (11)	12 (12)	10 (9)	9 (13)
Tibiofemoral lateral	22 (8)	10 (7)	12 (8)	5 (5)	10 (9)	5 (7)
Patellofemoral	93 (32)	55 (38)	38 (26)	28 (27)	40 (35)	24 (34)
Meniscal damage	55 (19)	20 (14)	35 (24)	25 (24)	17 (15)	9 (13)
Meniscal extrusion	68 (23)	32 (22)	36 (24)	21 (20)	31 (27)	14 (20)
Synovitis effusion	86 (29)	38 (26)	48 (32)	30 (29)	31 (27)	23 (33)
Hoffa synovitis	130 (44)	58 (40)	72 (49)	48 (46)	48 (42)	32 (46)
Popliteal cysts	83 (28)	42 (29)	41 (28)	33 (32)	32 (28)	16 (23)

**^a^**Data on BMI is missing for 6 individuals.

In the joint and compartment-specific analysis, the prevalence of cartilage damage was quite similar in the patellofemoral and tibiofemoral joints (199/294; 68% and 189/294; 64%, respectively; Table 2). The prevalence of cartilage damage was similar also in the medial and lateral compartments of the tibiofemoral joint (120/294; 41% and 127/294; 43%, respectively). On the other hand, osteophytes, bone marrow lesions, and subchondral cysts were observed more frequently in the patellofemoral joint.

### Prevalence in subjects without knee OA risk factors (reference cohort)

40 (87%) knees of subjects from the reference cohort had at least 1 type of structural abnormality. The most common MRI findings were cartilage damage (67%, CI 53–79), osteophytes (46%, CI 32–60), Hoffa synovitis (30%, CI 19–45), meniscal extrusions (22%, CI 12–36), and bone marrow lesions (35%, CI 23–49).

Cartilage damage, osteophytes, and bone marrow lesions were again observed more often in the patellofemoral joints than in the tibiofemoral compartments; the difference was biggest in the case of osteophytes (Table 3).

**Table 3.** Prevalence of MRI structural abnormalities in the right knees of the study subjects without knee OA risk factors (reference cohort). Values are frequency and (percentage)

MRI feature	Overall (n = 46)	Women (n = 27)	Men (n = 19)	BMI	
				< 25 (n = 26)	25–29 (n = 20)
Cartilage damage	31 (67)	21 (78)	10 (53)	18 (69)	13 (65)
Tibiofemoral	21 (46)	15 (56)	6 (32)	13 (50)	8 (40)
Tibiofemoral medial	15 (33)	12 (44)	3 (16)	10 (38)	5 (25)
Tibiofemoral lateral	11 (24)	8 (30)	3 (16)	6 (23)	5 (25)
Patellofemoral	26 (57)	19 (70)	7 (37)	16 (62)	10 (50)
Osteophytes	21 (46)	14 (52)	7 (37)	11 (42)	10 (50)
Tibiofemoral	5 (11)	4 (15)	1 (5)	4 (15)	1 (5)
Tibiofemoral medial	4 (9)	3 (11)	1 (5)	3 (12)	1 (5)
Tibiofemoral lateral	1 (2)	1 (4)	0 (0)	1 (4)	0 (0)
Patellofemoral	20 (43)	13 (48)	7 (37)	10 (38)	10 (50)
Bone marrow lesions	16 (35)	9 (33)	7 (37)	9 (35)	7 (35)
Tibiofemoral	5 (11)	3 (11)	2 (11)	3 (12)	2 (10)
Tibiofemoral medial	2 (4)	1 (4)	1 (5)	1 (4)	1 (5)
Tibiofemoral lateral	3 (7)	2 (7)	1 (5)	2 (8)	1 (5)
Patellofemoral	14 (30)	8 (30)	6 (32)	8 (31)	6 (30)
Subchondral cysts	9 (20)	7 (26)	2 (11)	7 (27)	2 (10)
Tibiofemoral	3 (7)	3 (11)	0 (0)	3 (12)	0 (0)
Tibiofemoral medial	1 (2)	1 (4)	0 (0)	1 (4)	0 (0)
Tibiofemoral lateral	2 (4)	2 (7)	0 (0)	2 (8)	0 (0)
Patellofemoral	6 (13)	4 (15)	2 (11)	4 (15)	2 (10)
Meniscal damage	5 (11)	2 (7)	3 (16)	2(8)	3 (15)
Meniscal extrusion	10 (22)	4 (15)	6 (32)	6 (23)	4 (20)
Synovitis effusion	8 (17)	7 (26)	1 (5)	4 (15)	4 (20)
Hoffa synovitis	14 (30)	8 (30)	6 (32)	10 (38)	4 (20)
Popliteal cysts	12 (26)	7 (26)	5 (26)	7 (27)	5 (25)

### Differences in the prevalence between the subjects with and without knee OA risk factors

The prevalence of any abnormality was higher (prevalence ratio adjusted for age, sex, and BMI 1.3 [CI 1.1–1.6]) in subjects with knee OA risk factors than in subjects without. In this study sample, with the exception of osteophytes and meniscal extrusions, all other MRI structural abnormalities were observed more often in subjects who had knee OA risk factors compared with those without. The prevalence ratios (CI; adjusted for age and sex) were for subchondral cysts 2.1 (1.2–3.8); bone marrow lesions 1.7 (1.2–2.6); meniscal tears 1.6 (0.7–3.8); synovitis–effusion 1.7 (0.9–3.2); Hoffa synovitis 1.4 (0.9–2.2); cartilage lesions 1.2 (1.0–1.5); popliteal cysts 1.1 (0.6–1.8); meniscal extrusion 1.1 (0.6–1.9); and osteophytes 1.0 (0.7–1.4) in subjects with knee OA risk factors compared with those without.

The prevalence of MRI-defined OA in the tibiofemoral joint was 23% (CI 19–28) in subjects with OA risk factors and 9% (CI 3–21) in those without. The corresponding proportions for the patellofemoral joint were 35% (CI 30–41) and 35% (CI 23–49), respectively.

The prevalence of MRI-defined tibiofemoral OA tended to be higher (prevalence ratio adjusted for age and sex 2.6 [CI 1.0–6.9]) in subjects with knee OA risk factors than in subjects without. The corresponding prevalence ratio for the patellofemoral joint was 1.0 (CI 0.7–1.5).

## Discussion

In contrast to several prior studies we examined the prevalence of knee joint structural features indicative of early knee OA on MRI in subjects aged 45 to 55 years without the evidence of knee OA on conventional radiographs (Hayes et al. 2005, Englund et al. 2008, Davies-Tuck et al. 2009, Javaid et al. 2010, Roemer et al. 2011, Guermazi et al. 2012, Hayashi et al. 2014, Sharma et al. 2014). At least 1 MRI-detected feature was found in 96% of the subjects with knee OA risk factors and in 87% of those without OA risk factors.

Cartilage damage was the most common MRI feature (82%), followed by BMLs (60%) and osteophytes (45%). Cartilage damage was equally highly prevalent in tibiofemoral and patellofemoral joints (64% and 68%, respectively), whereas BMLs and osteophytes were more common in patellofemoral joints in that age group (bone marrow lesions 47% and 31%; osteophytes 42% and 19% in patellofemoral and tibiofemoral joints, respectively). The prevalence of any abnormality was 32% higher in subjects with knee OA risk factors than in subjects without. Subchondral cysts, BMLs, meniscal tears, synovitis–effusion, Hoffa synovitis, cartilage damage, and popliteal cysts were observed more often in subjects with knee OA risk factors compared with the reference cohort. However, these differences reached statistical significance only in case of BMLs and subchondral cysts.

In the majority of prior knee OA studies the MRI features indicative of OA have been examined in older subjects with already radiographically evident knee OA (Raynauld et al. 2006, Bruyere et al. 2007, Madan-Sharma et al. 2008, Cibere et al. 2011, Crema et al. 2014, de Lange-Brokaar et al. 2016). Much less is known about the pre-radiographic stage of the disease when the basic radiographic features of knee OA—joint space narrowing and osteophytes—are not yet visible. In the pre-radiographic stage of OA, the structural features indicative of OA can be assessed by MRI as it has a greater sensitivity than radiography for the detection of OA-related bone and soft tissue changes (Felson et al. 1987, Bruyere et al. 2007). Still, there are concerns regarding its specificity.

According to the prior population-based observational study (Framingham Osteoarthritis Study) on 710 subjects with a mean age of 62 years and without radiographic knee OA, MRI detected lesions indicative of tibiofemoral OA were found in most subjects, regardless of knee pain. Among them 74% had osteophytes, 69% had cartilage damage, and 52% had BMLs (Guermazi et al. 2012). In another cohort study (OAI) on 849 subjects with a mean age of 60 years and without radiographic knee OA, 76% had cartilage damage, 61% bone marrow lesions, 21% meniscal tears, and 14% meniscal extrusion (Sharma et al. 2014). According to the Multicenter Osteoarthritis Study (MOST) on subjects aged 50–79 years without radiographic knee changes but at high risk of OA, again a high percentage of structural abnormalities was found by MRI (tibiofemoral or patellofemoral cartilage damage in 67–81%, bone marrow lesions in 55–75%, and osteophytes in as many as 99–100% of studied individuals) (Javaid et al. 2010). MRI features indicative of OA in our sample of radiographically normal subjects with a mean age of at least 10 years younger than in the prior studies were similarly highly prevalent.

We found that MRI-detected cartilage damage, osteophytes, BMLs, and subchondral cysts were more frequent in the patellofemoral than the tibiofemoral joint. Our findings are in accordance with the recent study by Lankhorst et al. who suggested that OA is more likely to start in the patellofemoral joint and then progress to the combined involvement of both the patellofemoral and tibiofemoral joint (Lankhorst et al. 2017). In another study the authors reported that isolated patellofemoral abnormalities were more common than isolated tibiofemoral abnormalities using MRI. Moreover, when mixed disease was present, the patellofemoral joint had more severe pathologies (Stefanik et al. 2013).

It has been reported that BMLs are strongly associated with knee pain (Felson et al. 2001, Yusuf et al. 2011) and might precede the development of radiographic knee OA (Javaid et al. 2010). In our study, BMLs were indeed significantly more prevalent in subjects with risk factors for knee OA compared with those without risk factors (60% and 35%, respectively), especially in the patellofemoral joints.

Our study has limitations. Only right knees were included in the analyses. Still, it is unlikely to affect our overall conclusion. Only central OAI readings for posteroanterior tibiofemoral joint radiographs were available at baseline. Further, we cannot exclude the potential for selection bias by including only subjects with MRI scans at all 4 time points, but is likely to affect both study cohorts (with and without risk factors) to a similar degree. Also, our findings cannot be generalized to the adult population younger than 45 years.

In summary, we found that MRI features indicative of knee OA are common in middle-aged subjects without radiographic knee OA. This was true irrespective of whether the knee OA risk factors were present or not. Subchondral cysts and BMLs were among the most frequent findings in subjects with multiple OA risk factors. The clinical importance of MRI findings related to OA remains vague. It is unclear to what extent these frequently encountered MRI findings represent early knee OA or whether they may perhaps be considered as part of “normal ageing” of the joint.

JK read the knee MRIs, participated in the analysis of data, interpreted the results, and drafted the first version of the manuscript. FZ measured meniscus extrusion, interpreted the results, and revised the manuscript for important intellectual content. AT analyzed the data, interpreted the results, and revised the manuscript. ME conceived the study, interpreted the results, and revised the manuscript. All authors approved the final version of the manuscript.

The authors would like to express their sincere gratitude to the OAI for the free use of public access data. They would like to acknowledge support from the Osteoarthritis Research Society International and the scholarship which made it possible for Jaanika Kumm to come to Lund University to perform the study.

*Acta* thanks Kirill Gromov for help with peer review of this study.
